# Chronic and Recurrent Depression in Primary Care: Socio-Demographic Features, Morbidity, and Costs

**DOI:** 10.1155/2012/316409

**Published:** 2012-06-06

**Authors:** Elaine M. McMahon, Marta Buszewicz, Mark Griffin, Jennifer Beecham, Eva-Maria Bonin, Felicitas Rost, Kate Walters, Michael King

**Affiliations:** ^1^Research Department of Primary Care and Population Health, University College London, Upper Third Floor, Royal Free Hospital, Rowland Hill Street, London NW3 2PF, UK; ^2^Personal Social Services Research Unit, London School of Economics and Political Science, Houghton Street, London WC2A 2AE, UK; ^3^Personal Social Services Research Unit, University of Kent, Cornwallis Building, Canterbury, Kent CT2 7NF, UK; ^4^Research Department of Mental Health Sciences, University College London, Royal Free Campus, Rowland Hill Street, London NW3 2PF, UK

## Abstract

*Background*. Major depression is often chronic or recurrent and is usually treated within primary care. Little is known about the associated morbidity and costs. *Objectives*. To determine socio-demographic characteristics of people with chronic or recurrent depression in primary care and associated morbidity, service use, and costs. *Method*. 558 participants were recruited from 42 GP practices in the UK. All participants had a history of chronic major depression, recurrent major depression, or dysthymia. Participants completed questionnaires including the BDI-II, Work and Social Adjustment Scale, Euroquol, and Client Service Receipt Inventory documenting use of primary care, mental health, and other services. *Results*. The sample was characterised by high levels of depression, functional impairment, and high service use and costs. The majority (74%) had been treated with an anti-depressant, while few had seen a counsellor (15%) or a psychologist (3%) in the preceding three months. The group with chronic major depression was most depressed and impaired with highest service use, whilst those with dysthymia were least depressed, impaired, and costly to support but still had high morbidity and associated costs. *Conclusion*. This is a patient group with very significant morbidity and high costs. Effective interventions to reduce both are required.

## 1. Introduction

In the UK, clinically significant or major depression affects between 5% and 10% of people at any time, with most being treated within general practice [1]. The annual cost of depression has been estimated to be over *£*9 billion in England [2], with more than 100 million working days lost and over 2,500 deaths due to depression in 2000 [2].

Major depression is often a chronic or recurrent disorder, with an estimated 80% of people experiencing at least one recurrence, although one primary care study has reported recurrence rates as low as 40% after a first episode [3]. Approximately 12% follow a chronic course [4]. Results from other studies indicate that only 50% of those with major depression will have recovered at one year [5]. The risk of recurrence increases with each successive episode [6].

Primary care populations with chronic or recurrent depression, although clinically important, are rarely investigated as a distinct patient group [7]. Past work has shown that chronicity is associated with high mortality, greater psychological and social morbidity, high use of primary care services [8], and high social and financial costs [3]. However, we have little detailed information on the specific morbidity, functional impairment, health service use or costs of chronic or recurrent depression in primary care settings.

In this study, our aims were to examine socio-demographic characteristics, morbidity, service use, and associated costs for three main clinical groups of people with depression. Our sample comprised people looked after in primary care who were diagnosed with chronic major depression, recurrent major depression, and dysthymia. We addressed three main questions as follows.

1. What were the socio-demographic characteristics of people in these three groups, and were there differences between the groups?
2. Were there differences in the associated morbidity?
3. What services were used by these three groups, and what were the associated costs?

## 2. Methods

### 2.1. Recruitment

A total of 558 participants were recruited between November 2007 and July 2008 from 42 GP practices in England, Scotland, and Northern Ireland. The aim was to recruit a representative sample of practices from throughout the UK, including urban and rural areas and areas with diverse ethnic populations. Participants were identified to be part of multi-centre study to test whether regular proactive contact with a practice nurse would benefit people with chronic or recurrent depression [9]. Patients were eligible if they were over the age of 18, had a recent history of chronic or recurrent major depression or dysthymia based on the Composite International Diagnostic Interview (CIDI), and their symptoms indicated at least mild depression (scoring 14 or higher on the Beck Depression Inventory; BDI-II). Patients with impaired cognitive function, current psychotic symptoms, or incapacitating drug or alcohol dependence were excluded. All patients who met eligibility criteria and who consented to participate were included in the study. Recruitment procedures, inclusion/exclusion criteria, and outcome measures used have been fully described elsewhere [9]. The findings reported here are based on pooled data collected at baseline from both intervention and control participants before the intervention began.

### 2.2. Measures

Prior to enrolment, participants were interviewed by the practice/research nurse to check eligibility. The nurses had been trained to conduct the recruitment interview. Research questionnaires were completed by eligible participants immediately after the interview and prior to randomisation.

The CIDI was administered by the nurse to check eligibility. Eligible participants met criteria for one of three DSM-IV diagnoses as described in Box 1 [10]. 

The self-report questionnaires completed included assessment of severity of depressive symptoms using the BDI-II, a widely used 21-item questionnaire [11]. Functional impairment was measured using the Work and Social Adjustment Scale (WSAS), a 5-item measure of impairment attributable to an identified problem (e.g., depression) [12]. Service use was assessed using the Client Service Receipt Inventory (CSRI), a self-complete record of demographic data, medication, and health and community service use for the three months prior to recruitment [13].

**Cost Calculations**

The costs of service use for each person were calculated by identifying an appropriate unit cost for each contact and multiplying it by the number of contacts each person recorded on the CSRI. For most hospital, mental health and primary care services as well as social service interventions, unit costs were drawn from publicly available sources [14, 15]. The remainder was taken from previous studies or estimated using an equivalent method [16]. Where the number of service contacts was missing, the mean for the whole group or a minimum of one contact was used. All costs are presented in 2008 prices.

### 2.3. Data Analysis

Descriptive statistics were produced for socio-demographic characteristics, BDI II and WSAS, by each diagnostic group. Findings are presented as percentages for categorical variables and means, and standard deviations are used for continuous variables as the data were found to be approximately normally distributed.

The proportion of people using selected services and the contact rates are reported by diagnostic group. Costs are compared between diagnostic groups using *t*-tests with 1,000 bootstrap replications [17] using STATA version 10.0 and SPSS version 17.0 software.

## 3. Results

### 3.1. Characteristics of the Sample

#### 3.1.1. Socio-Demographic Characteristics

Table 1 reports the demographic and diagnostic characteristics of the study sample; 54% of participants met criteria for recurrent major depression, 30% for chronic major depression, and 16% for dysthymia. The average age of the sample was 48 years, and 75% were female. Two thirds were owner occupiers of their homes. Just under half were in paid employment. A greater proportion of those with dysthymia were married or cohabiting (66%) compared to those with chronic major depression (53%) or recurrent depression (54%). Over half of those with recurrent depression were in paid employment (56%), compared to just 35% of those with chronic major depression and 39% of those with dysthymia. 

#### 3.1.2. Depression and Functional Impairment

Tables 2 and 3 report participants' scores on measures of depression and functional impairment by diagnostic group. Higher scores on the BDI-II and WSAS scales indicate higher levels of depression and functional impairment. 62% of the entire sample were categorised as severely depressed [11] and 61% categorised as moderately or severely functionally impaired [12]. Those with chronic major depression had the highest mean BDI II score, and 76% of this group were severely depressed, compared with 57% of those with recurrent depression and 54% with dysthymia. Participants with chronic major depression also had the highest mean WSAS impairment score with 69% of this group at least moderately impaired. 

#### 3.1.3. Service Use and Costs

Costs were available for 549 participants, although we excluded one person who remained in hospital for the full period as no community or primary care services were used.  Table 4 identifies service use patterns between the groups, showing in detail the use of GP, practice nurse, and mental health services and conflating others into five main categories. In the previous 3 months, 63% of the chronic major depression group had consulted a GP for depression, a higher proportion than either the recurrent depression or dysthymia groups. However, the dysthymia group had the highest proportion who reported any primary care consultations for all reasons in the past three months (89%). A minority of the total sample had seen a counsellor (15%) or a psychologist (3%).

Across the whole sample, a wide range of services was used although, with the notable exception of primary care, most services were only used by one or two people. Very few people across the whole sample had seen a secondary care mental health professional, either a psychiatrist (5%) or psychologist (3%), while around a sixth (15%) had had contact with a practice counsellor. Despite the differences in severity of depression and levels of functional impairment, a similar proportion of people in each of the three groups had used each of the services with two exceptions.  Table 4 shows that, compared to the other groups, those with dysthymia were less likely to be in contact with any mental health services and a higher proportion those with recurrent depression had seen an alternative (complementary) therapist.

 The majority of the sample (74%) had been prescribed anti-depressant medication in the previous 3 months, including 80% of those with chronic major depression, 72% of those with recurrent depression, and 70% with dysthymia. Anti-depressants were by far the most commonly prescribed medication in this population.

Table 5 shows the costs of support for each diagnostic group over the 3-month period. Hospital costs account for the highest proportion of total costs for the total sample and also within each diagnostic group (around a third), followed by primary care and mental health services. Cost differences between groups generally reflect the service use patterns (Table 4). Average costs for primary care and other health and community services were similar in each group.

Bootstrapped *t*-tests found significantly higher mean costs for the recurrent depression and chronic major depression groups compared to those with dysthymia for mental health services, alternative therapy, and total costs. Those with recurrent depression also had higher costs for alternative therapy and significantly lower costs for “other medications” than those with chronic major depression.

## 4. Discussion

Three groups were identified among this sample of primary care patients based on the DSM-IV diagnostic criteria for chronic major depression, recurrent depression, or dysthymia. The sample as a whole was characterised by very high levels of depressive symptoms and functional impairment, and the majority had been prescribed anti-depressants in the preceding three months. Those with chronic major depression were the most depressed and impaired of the three groups. Compared to the other two groups, they had the highest costs for mental health service use and the costs for their full service package were also highest (final row, Table 5). People with dysthymia were the least depressed and had less severe functional impairment; their total service costs were lowest, as was the proportion in contact with mental health services.

 All three groups made considerable use of GPs, with on average slightly more than one GP consultation for depression and more than one consultation for other reasons in the previous three months. If the three months prior to interview are representative of the full year, these suggest higher contact rates than in the population as a whole. National data show women have an average of five GP appointments per year for all reasons and men have four [18].

Levels of depression were much higher among our sample than reported in another primary care study of recurrent depression which used the BDI-II as an outcome measure [7], but this may be due to the eligibility criterion for this study (above 14 on the BDI-II) which was intended to ensure inclusion of those with at least minor depression at the point of recruitment. However, our findings support previous research showing reductions in functioning and well-being for depressed patients that equal or exceed those of patients with other chronic illnesses [19].

The percentage of participants in paid employment was lower than reported in other studies of primary care patients with depression [20], reflecting the greater impairment likely to be associated with chronic or recurrent depression. In line with other findings, the participants with chronic major depression were least likely to be in paid employment. In our sample, men reported significantly higher functional impairment than women, but the severity of their depression was similar. Notably, more than three-quarters of participants had received a prescription for anti-depressants within the past three months, but most continued to have very high levels of depression and associated functional impairment, which suggests that anti-depressant therapy is far from optimal in this group. Around a quarter had seen a mental health professional in the past three months, but most of these contacts were with a counsellor.

A limitation of this study is that participants were specifically recruited for a research study and may not therefore be representative of the whole population of people with chronic or recurrent depression in primary care. There were fewer GP practices from inner city areas participating in the study, and our sample therefore under-represents populations from these areas. This may affect the generalisability of our findings to more ethnically and socially diverse inner city populations. Nonetheless, our large study sample comprises a rigorously selected group of patients and indicates the high levels of morbidity and functional impairment associated with chronic major depression, recurrent major depression, and dysthymia, as well as the differences between these three diagnostic groups.

People with chronic/recurrent major depression and dysthymia form clinically important patient groups for primary care practitioners. Nearly two-thirds of our sample had severe depressive symptoms and high functional impairment, despite the majority receiving anti-depressant treatment. Most were being treated entirely in a primary care setting where regular followup and review may be lacking [21, 22]. The chronic nature of their problems and high rates of attendance in primary care suggests they are particularly challenging for GPs to work with. Moreover, one in four of those in our study with chronic major or recurrent depression had also seen a mental health professional, at least one in three of the whole sample had attended a hospital-based service, and one in four had contact with at least one other health or community care service during the three months prior to the start of the main study. Further data collected prospectively for this sample within the over-arching trial will allow us to test whether a structured proactive practice nurse-led intervention is an effective form of intervention for this group.

## Figures and Tables

**Box 1:**
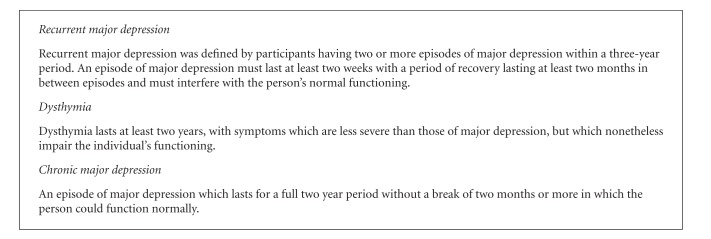
DSM-IV diagnoses included in the ProCEED study.

**Table 1:** Socio-demographic and diagnostic characteristics.

	Diagnosis			
	Chronic major depression	Recurrent depression	Dysthymia	Total
Total sample	164 (29.8%)	297 (54%)	89 (16.2%)	550
*Diagnosis by gender *				
Male	51 (31.1%)	57 (19.2%)	31 (34.8%)	139 (25.3%)
Female	113 (68.9%)	240 (80.8%)	58 (65.2%)	411 (74.7%)

Age				

Mean (SD) [range]	50 (14) [18–85]	46 (12) [20–63]	52 (13) [23–77]	48 (13) [18–85]

Ethnic group (*n* = 543)				

White	154 (96.3%)	283 (96.0%)	83 (94.3%)	520 (95.8%)
Other	6 (3.1%)	12 (4.0%)	5 (5.7%)	23 (4.2%)

Marital status (*n* = 546)				

Married/cohabiting	86 (53.1%)	160 (54.2%)	59 (66.3%)	305 (55.9%)
Divorced/separated/widowed/single	76 (46.9%)	135 (45.8%)	30 (33.7%)	241 (44.1%)

Living situation (*n* = 546)				

Partner/children	113 (70.2%)	216 (72.7%)	67 (76.1%)	396 (72.5%)
Other	48 (29.8%)	81 (27.3%)	21 (23.9%)	150 (27.5%)

Housing (*n* = 541)				

Owner occupied	103 (64.8%)	196 (66.7%)	64 (72.7%)	363 (67.1%)
Rented/temporary accommodation	56 (35.2%)	98 (33.3%)	24 (27.3%)	178 (32.9%)

Occupation (*n* = 545)				

Paid employment	57 (35.2%)	165 (55.9%)	34 (38.6%)	256 (47.0%)
Long term Sick/retired/homemaker/unemployed/other	105 (64.8%)	130 (44.1%)	54 (61.4%)	289 (53.0%)

**Table 2:** Severity of depression by diagnostic group.

	Diagnosis			
	Chronic major depression (*n* = 164)	Recurrent depression (*n* = 297)	Dysthymia (*n* = 89)	Total (*n* = 550)
BDI-II total score				

Mean (SD)	36.0 (10.7)	31.4 (9.9)	29.76 (8.6)	32.52 (10.2)
Range	14–60	14–57	14–49	14–60

BDI-II depression severity				

Mild depression	11 (6.7%)	39 (13.1%)	11 (12.4%)	61 (11.1%)
Moderate depression	29 (17.7%)	88 (29.6%)	30 (33.7%)	147 (26.7%)
Severe depression	124 (75.6%)	170 (57.2%)	48 (53.9%)	342 (62.2%)

**Table 3:** Severity of functional impairment by diagnostic group.

	Diagnosis			
	Chronic major depression (*n* = 163)	Recurrent depression (*n* = 296)	Dysthymia (*n* = 88)	Total (*n* = 547)
WSAS total score				

Mean (SD)	24.97 (9.7)	21.41 (9.4)	20.38 (8.4)	22.3 (9.5)
Range	0–40	0–40	3–38	0–40

WSAS impairment severity				

Subclinical impairment	11 (6.7%)	40 (13.5%)	10 (11.4%)	61 (11.2%)
Significant impairment	39 (23.9%)	84 (28.4%)	29 (33.0%)	152 (27.8%)
Moderately severe or worse impairment	113 (69.3%)	172 (58.1%)	49 (55.7%)	334 (61.1%)

**Table 4:** Service use over 3 months, by diagnostic group.

Service^a^	Chronic major depression (*n* = 163)		Recurrent depression (*n* = 296)		Dysthymia (*n* = 89)		Total (*n* = 548)	
	% using service	Mean number contacts	% using service	Mean number contacts	% using service	Mean number contacts	% using service	Mean number contacts
GP (depression)	63%	1.16	57%	1.16	58%	1.16	59%	1.16
GP (other reason)	61%	1.38	57%	1.16	67%	1.29	60%	1.25
Practice nurse (depression)	9%	0.12	5%	0.06	9%	0.12	7%	0.09
Practice nurse (other)	33%	0.51	31%	0.49	28%	0.40	31%	0.49

Any primary care	85%	—	88%	—	89%	—	87%	—

Psychiatrist	4%	0.13	5%	0.08	4%	0.07	5%	0.09
Psychologist	7%	0.31³	1%	0.03³	3%	0.06	3%	0.11
Counsellor	16%	0.93²	16%	0.92*¹*	9%	0.33*¹*	15%	0.82

Any mental health contacts	28%	—	28%	—	16%	—	26%	—

Any hospital services	40%	—	35%	—	37%	—	37%	—

Any alternative therapy	7%	0.21²	11%	0.78*¹*	1%	0.01^1,2^	9%	0.48

Any other health and community services	26%	—	25%	—	27%	—	26%	—

Anti-depressants	80%	—	72%	—	70%	—	74%	—

Other medications	77%	—	63%	—	79%	—	70%	—

^a^Any primary care includes all GP and practice nurse contacts, other primary care services. Any mental health services include psychiatrist, psychologist, counsellor, psychotherapy, psychiatric community nurse. Any hospital services include inpatient and outpatient services for depression or other reason, A&E or minor injuries unit. Any alternative therapy includes, for example, hydrotherapy, spiritual healing. Any other health and community services include community health services, social work and other social care, self-help and support services, support provided by the voluntary sector.

^1^Significant difference between dysthymia and recurrent depression groups.

^2^Significant difference between dysthymia and chronic major depression groups.

^3^Significant difference between recurrent depression and chronic major depression groups.

**Table 5:** Average costs over three months, by service category and diagnostic group.

Service category^a^	Chronic major depression (*n* = 163)	Recurrent depression (*n* = 296)	Dysthymia (*n* = 89)	Total (*n* = 548)
	Mean costs (SD)	Mean costs (SD)	Mean costs (SD)	Mean costs (SD)
Primary care	*£*96 (80)	*£*88 (77)	*£*92 (67)	91 (77)
Mental health	*£*147² (401)	*£*107*¹* (317)	*£*40^1,2^ (136)	105 (325)
Hospital	*£*176 (463)	*£*154 (520)	*£*107 (230)	153 (467)
Alternative therapy	*£*9^2,3^ (48)	*£*33^1,3^ (235)	*£*0^1,2^ (4)	21 (175)
Other health and community services	*£*67 (232)	*£*54 (213)	*£*57 (218)	58 (219)
Anti-depressants	*£*12 (20)	*£*12 (21)	*£*11 (19)	12 (20)
Other medications	*£*17³ (13)	*£*12^1,3^ (14)	*£*15^1^ (15)	14 (15)

Total costs	*£*523² (784)	*£*460^1^ (787)	*£*322^1,2^ (386)	457 (738)

^a^See Table 4 for services included in each category.

^1^Significant difference between dysthymia and recurrent depression groups.

^2^Significant difference between dysthymia and chronic major depression groups.

^3^Significant difference between recurrent depression and chronic major depression groups.
